# Social Alienation in Schizophrenia Patients: Association with Insula Responsiveness to Facial Expressions of Disgust

**DOI:** 10.1371/journal.pone.0085014

**Published:** 2014-01-22

**Authors:** Christian Lindner, Udo Dannlowski, Kirsten Walhöfer, Maike Rödiger, Birgit Maisch, Jochen Bauer, Patricia Ohrmann, Rebekka Lencer, Pienie Zwitserlood, Anette Kersting, Walter Heindel, Volker Arolt, Harald Kugel, Thomas Suslow

**Affiliations:** 1 Department of Psychiatry, University of Münster, Münster, Germany; 2 Department of Psychiatry, University of Marburg, Marburg, Germany; 3 Klinik am Schlossgarten, Dülmen, Germany; 4 Institute of Psychology, University of Münster, Münster, Germany; 5 Department of Psychosomatic Medicine and Psychotherapy, University of Leipzig, Leipzig, Germany; 6 Department of Clinical Radiology, University of Münster, Münster, Germany; Ecole Normale Supérieure, France

## Abstract

**Introduction:**

Among the functional neuroimaging studies on emotional face processing in schizophrenia, few have used paradigms with facial expressions of disgust. In this study, we investigated whether schizophrenia patients show less insula activation to macro-expressions (overt, clearly visible expressions) and micro-expressions (covert, very brief expressions) of disgust than healthy controls. Furthermore, departing from the assumption that disgust faces signal social rejection, we examined whether perceptual sensitivity to disgust is related to social alienation in patients and controls. We hypothesized that high insula responsiveness to facial disgust predicts social alienation.

**Methods:**

We used functional magnetic resonance imaging to measure insula activation in 36 schizophrenia patients and 40 healthy controls. During scanning, subjects passively viewed covert and overt presentations of disgust and neutral faces. To measure social alienation, a social loneliness scale and an agreeableness scale were administered.

**Results:**

Schizophrenia patients exhibited reduced insula activation in response to covert facial expressions of disgust. With respect to macro-expressions of disgust, no between-group differences emerged. In patients, insula responsiveness to covert faces of disgust was positively correlated with social loneliness. Furthermore, patients' insula responsiveness to covert and overt faces of disgust was negatively correlated with agreeableness. In controls, insula responsiveness to covert expressions of disgust correlated negatively with agreeableness.

**Discussion:**

Schizophrenia patients show reduced insula responsiveness to micro-expressions but not macro-expressions of disgust compared to healthy controls. In patients, low agreeableness was associated with stronger insula response to micro- and macro-expressions of disgust. Patients with a strong tendency to feel uncomfortable with social interactions appear to be characterized by a high sensitivity for facial expression signaling social rejection. Given the associations of insula responsiveness to covert disgust expression with low agreeableness in healthy individuals, insula responsiveness to expressions of disgust might be in general a neural marker of the personality trait of agreeableness.

## Introduction

The last decades have seen a rise of studies investigating abnormalities in the processing of facial affect in schizophrenia patients. Most studies agree in that patients show significant deficits in facial affect recognition [1], [2], [3]. On a neural level, these deficits have mostly been related to amygdala dysfunction, with some studies reporting amygdala under-recruitment in response to emotional faces [4], [5] and others reporting amygdala over-recruitment [6], [7].

The major part of neuroimaging studies in this field has used experimental paradigms including fearful faces. This seems plausible as the emotion recognition deficit for fearful faces is particularly pronounced in schizophrenia patients [1]. Importantly, the processing of fearful faces is closely related to the amygdala [8], [9], [10].

On the contrary, only a few neuroimaging studies have focused on facial stimuli displaying the emotion of disgust. The processing of facial expressions of disgust has been associated with another one particular brain region, namely the insula [11], [12], [13]. To our knowledge, only one study analyzed the neural response to expressions of disgust in schizophrenia patients [14]. The authors used an emotion recognition paradigm and found a slight hypoactivation of the insular cortex in patients. A major shortcoming of this study is the small sample size of schizophrenia patients (N = 10). Other studies combined disgust with other negative facial emotions in their statistical analyses [15], [6] so that no distinct interpretation can be drawn with respect to the processing of facial disgust in schizophrenia.

The neglect of disgust in neuroimaging studies on emotional face processing in schizophrenia is regrettable. First of all, behavioral studies have shown that patients manifest pronounced impairments in the recognition of facial expressions of disgust [1]. The disgust recognition deficit in patients might not be explained by amygdala dysfunction, as the amygdala is not specifically involved in processing disgust [13]. Rather, deficits in disgust recognition have been attributed to insula dysfunction [14], [16].

Another aspect warranting a closer look on disgust resides in the social dimension of this emotion. According to Rozin et al. [17] the emotion of disgust evolved as a rejection response to bad tastes and developed into a more abstract form of general rejection. In this vein, it also subserves a function of protecting the individual from interpersonal “contamination”. Sherman and Haidt [18] view disgust as an affective mechanism for dampening motivations for social interactions. They suggest that this emotion may even have a dehumanizing function in social relationships, as the objects of disgust are excluded from moral concern. Facial expressions of disgust can represent a signal of social rejection, indicating a “request to go away” [19]. Recent neuroimaging research has delivered evidence in support of this perspective, as perceiving disgust in others and experiencing social rejection share a similar neural correlate. The insula, which is implicated in the processing of facial expressions of disgust, also seems to be involved in the experience of social rejection and social pain [20], [21].

In the case of schizophrenia patients, the social dimension of disgust is particularly interesting. On the one hand, patients often experience stigmatization because of the disease and are thus confronted with social rejection more often than healthy people [22]. On the other hand, Suslow et al. [23] have found that schizophrenia patients tend to experience more feelings of disgust in everyday live and argue that this emotion might protect patients from interpersonal infringements. Against this background, the emotion of disgust might play a role in the development and maintenance of social alienation in patients.

In everyday communication, negative emotions are often not expressed overtly but rather occur in the form of micro-expressions, i.e. subtle and very brief expressions of an emotion [24]. These micro-expressions fulfill a regulatory function in social interactions [25]. Importantly, perceptual sensitivity to micro-expressions differs between individuals [26] and may in some cases represent an interpersonal advantage [27]. Conversely, sensitivity to micro-expressions of disgust – subtle expressions of social rejection – might arguably be a burden in social interactions and foster interpersonal distancing and social alienation.

The first aim of the present study was to investigate the neural correlates of the processing of micro-expressions and macro-expressions of disgust in schizophrenia patients. To our knowledge, no neuroimaging study has ever investigated the processing of micro-expressions of disgust in schizophrenia. We used a passive viewing paradigm with stimuli designed to mimic micro- and macro-expressions of facial emotions (disgust, happy, fearful and neutral). For the purpose of the present study, the paradigm was selectively analyzed, as we focused on disgusted face stimuli and used happy faces stimuli as a control condition. A particular strength of passive viewing paradigms consists in the minimal cognitive load as cognitive load might attenuate the processing of emotional faces [7]. In order to simulate micro-expressions, we applied a backward masking technique where faces expressing disgust (and other basic emotions) were presented at the perceptual threshold and masked by neutral face stimuli (see Methods). Based on preliminary findings of Phillips et al. [14], we hypothesized that patients would show lower insula activation than controls during the viewing of masked and unmasked disgust faces. As most studies on emotional face processing in schizophrenia have focused on the amygdala as core region of interest, we included also the amygdala in our between-group analysis.

A second aim of the study was to test whether the neural responsiveness to facial expressions of disgust is related to social alienation in patients and in healthy controls. As outlined above, heightened insula responsiveness to expressions of disgust might be associated with increased perceptions of social rejection and thus prompt withdrawal and alienation. We assessed the degree of social loneliness and the personality trait agreeableness as indices of social alienation in our sample. Social loneliness was chosen as a direct measure of alienation, and agreeableness was chosen as a social personality trait reflecting individual differences in trusting cooperation and the tendency to be comfortable with social interactions. It has been shown for schizophrenia patients, that the frequency of social interaction was predicted by higher levels of agreeableness [28]. We expected that insula activation to disgust faces would be positively correlated with social loneliness and negatively correlated with agreeableness. As a control condition, we used happy facial expressions and expected that insula responsiveness to happy faces would not be correlated with neither social loneliness nor agreeableness in both groups. Finally, we tested whether these associations would be different for patients versus controls and for covert versus overt expressions of disgust.

## Methods

### Ethics statement

The study was conducted in accordance with the Declaration of Helsinki as revised in 1989 and was approved by the Ethics Committee of the University of Münster. After a complete description of the study, written informed consent was obtained from all subjects. Of the 38 psychiatric in-patients participating in the study, none were impaired in their ability to consent. This was established by the attending clinicians and senior physicians who ensured that the patients were clinically stable and able to correctly judge the potential risks and benefits of their participation. During the study description, it was ascertained that all participants could understand the aims of the study.

### Subjects

The original sample encompassed 38 schizophrenia patients and 42 healthy controls aged between 18 and 55 years. Participants received a financial compensation. For all participants, exclusion criteria were a history of neurological disease, severe head trauma causing a loss of consciousness, substance abuse during the last six months and the usual magnetic resonance imaging contraindications. All subjects had normal or corrected-to-normal vision. Control subjects were thoroughly investigated by trained psychologists and were free of any lifetime history of psychiatric disorders, as diagnosed by the Structured Clinical Interview for DSM-IV, Axis I disorders (SCID [29]). Patients' diagnoses were established by senior psychiatrists and confirmed by trained interviewers with the SCID interview. During the patient interview, special attention was given to affective disorders to exclude a current depressive episode in study patients.

To assess subjects' social loneliness, we administered the multidimensional loneliness questionnaire (Multidimensionaler Einsamkeitsfragebogen; MEF [30]). The multidimensional loneliness questionnaire encompasses 37 items (single item score range from 1 to 5) and covers three dimensions of loneliness. The ‘social loneliness scale’, which represents the focus in the present study, measures feelings of social isolation (15 items). The ‘emotional loneliness scale’ measures the ability to maintain satisfactory intimate relationships (12 items). The third scale covers the “inability to be alone” and is a measure of subjective distress caused by loneliness (10 items). To assess subjects' agreeableness, we used the agreeableness scale from the 5-factor personality questionnaire NEO-FFI (12 items, single item score range from 0 to 4) [31]. For both the social loneliness scale and the agreeableness scale, scale scores were calculated by adding up the single item scores.

In addition, all patients were administered a structured protocol of the Scale for the Assessment of Negative Symptoms (SANS [32]) and the Scale for the Assessment of Positive Symptoms (SAPS [33]). For all participants, depressivity was measured with the Beck Depression Inventory (BDI [34]), and trait anxiety was measured with the State-Trait-Anxiety-Inventory (STAI, trait version [35]). Verbal intelligence was estimated by the Multiple-Choice-Vocabulary-Intelligence-Test [36].

Due to excess motion, four subjects (2 patients, 2 controls) had to be excluded from the study. The final sample encompassed 40 healthy controls as well as 36 patients. Table 1 lists the sociodemographic and clinical data of all participants. Of the 36 patients, all received second-generation antipsychotics, and three received additional typical antipsychotics. Fourteen patients were taking concomitant antidepressant medication; four were taking anticonvulsives; and one was taking lithium.

**Table 1:** Sociodemographic and clinical data.

	Patients	Controls	p
Age	30.8±7.9 (18–51)	29.5±8.3 (19–49)	0.49
Education years	13.1±2.6 (8–18)	14.6±2.4 (9–18)	0.015
Parental education years^1^	14.5±3.0 (9–18)	15.0±2.6 (11–18)	0.42
Sex (m/f)	22/14	27/13	0.64
Handedness (right/left)	34/2	38/2	1.000
Verbal intelligence^2^	105.6±13.7 (77–136)	113.5±11.8 (95–136)	0.008
BDI	12.7±6.9 (0–28)	2.4±2.8 (0–10)	<0.001
STAI-T	46.4±9.5 (25–71)	30.6±6.7 (22–46)	<0.001
SANS^3^ – flat affect	2.1±1.2 (0–5)		
SANS – alogia	1.7±1.1 (0–4)		
SANS – apathy	2.2±0.6 (1–4)		
SANS – anhedonia	2.1±1.0 (0–4)		
SANS – attention	1.9±0.8 (0–4)		
SAPS – hallucinations	0.4±0.6 (0–2)		
SAPS – delusions	1.2±1.0 (0–4)		
SAPS – bizarre behavior	0.7±0.8 (0–2)		
SAPS – formal thought disorder	1.6±1.2 (0–4)		
Years of illness	6.8±5.6 (0.5–19.0)		

Sociodemographic and clinical data of patients and controls (patients: N = 36; controls: N = 40) and clinical data of patients; mean ± SE (range); p: significance of two sample t-test/chi-square-test between patients and controls.

^1^ Education years of parent with the highest degree. Data missing for one patient.

^2^ Assessed with the Mehrfachwahl-Wortschatz-Intelligenztest (MWT-B [36]).

^3^ All SANS and SAPS scores represent global ratings of the symptom.

### Paradigm

We used a passive viewing paradigm as described in other studies from our research group [37], [38]. The paradigm consisted of two sequences in which a sequence with masked emotional facial expressions was followed by a sequence with unmasked expressions. For the paradigm as a whole, the facial expressions displayed were fear, disgust, happiness and neutral. Facial stimuli consisted of images selected from the Karolinska Directed Emotional Faces (KDEF) catalogue [39]. Stimuli represented the faces of male and female actors, and different actors were chosen for the two sequences (10 male and 10 female actors for each sequence).

**Figure 1:**
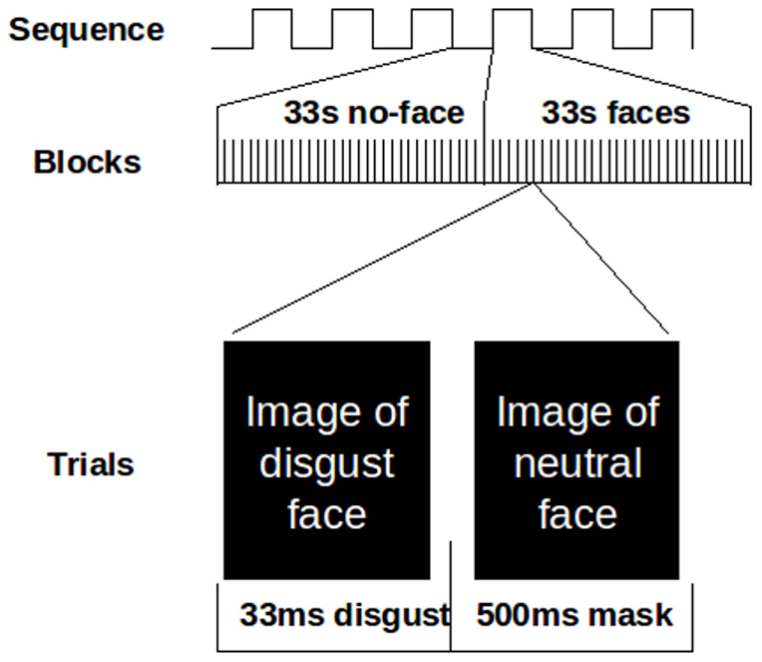
Illustration of paradigm. In the micro-expressions sequence displayed here, emotional expressions were masked by neutral expressions. In the macro-expressions sequence, facial expressions were presented for 533 ms. Both sequences consisted of alternating blocks of face stimuli and no-face stimuli. In each block, facial stimuli were presented in a randomized order.

In both sequences, subjects were instructed to attentively watch and memorize the facial stimuli. Subjects were presented with 33 s blocks of a facial expression category (fear, disgust, happy, neutral) or a no-face stimulus (skin-colored semi-oval). The no-face stimulus was used as a general baseline condition to assess task effects. In the first sequence (masked expressions), facial prime stimuli were presented for 33 ms and followed by a 500 ms mask depicting a neutral face of the same actor (Fig. 1). In the second sequence (unmasked expressions), subjects viewed faces for 533 ms. Each emotion block was followed by a no-face block and presented twice, resulting in a presentation time of 8 min and 48 sec per sequence (total time: 17 min and 36 sec). In both sequences, each facial expression category was thus presented for a total of 66 s. The order of the blocks was counterbalanced across subjects according to a Latin square design.

After scanning, subjects were administered a structured protocol exploring awareness of masked stimuli. They were questioned as to whether they had noticed anything out of the ordinary during the first sequence and whether they had perceived anything before the neutral faces. Subjects reporting subjective awareness of the masked facial stimuli completed a forced-choice questionnaire concerning the emotional qualities they perceived.

### fMRI methods

Images were projected to the rear end of the scanner (SharpXG-PC10XE with additional RF shielding (Covilex), Magdeburg, Germany). T2* functional data were acquired with a 3-Tesla scanner (Gyroscan Intera 3.0T, Philips Medical Systems, Best, NL) using a single-shot echoplanar sequence with parameters selected to minimize distortion in the regions of central interest while retaining adequate S/N and T2* sensitivity. Volumes consisting of 36 axial slices were acquired (matrix 64 * 63, resolution 3.6 mm * 3.6 mm * 3.5 mm; repetition time  = 2.5 s, echo time  = 35 ms, flip angle  = 90°) 212 times in a block design.

Functional imaging data were motion-corrected, using a set of six rigid body transformations determined for each image, spatially normalized to the standard Montreal Neurological Institute (MNI) template with a voxel size of 2 mm edge length, and smoothed (Gaussian kernel, 8 mm FWHM) using Statistical Parametric Mapping (SPM5, Wellcome Department of Cognitive Neurology, London, UK). Statistical analysis was performed by modeling the different conditions (fear, happy, disgust, neutral) as variables within the context of the general linear model convolved with a standard hemodynamic response function. Fixed-effect analyses were performed at the individual level to generate individual contrast maps, and random effect analyses were performed at the group-level. On a single subject level, contrast images were calculated comparing each emotion's face block (fear, happy and disgust) with the neutral face baseline.

### Second-Level fMRI analysis

In the scope of the present paper, we focus on the disgust versus neutral contrasts. The happy versus neutral contrasts were used as a control condition for the correlational analysis. Standard univariate group-level statistics were calculated using SPM5. The insula and the amygdala were chosen as the a priori regions of interest (ROI) for the assessment of task effects and for the between-group analysis and the insula was chosen as a priori ROI for the correlational analyses. The ROIs were defined according to Tzourio-Mazoyer et al. [40], and the insula and amygdala masks were created by means of the WFU PickAtlas [41]. To control for multiple statistical testing, we maintained a cluster-level false-positive detection rate at p<0.05 using an uncorrected voxel threshold of p<0.05 with a cluster extent (k voxels) empirically determined by Monte Carlo Simulations (n = 1000 iterations) for the bilateral insula and the bilateral amygdala. This was performed by means of the AlphaSim procedure, which accounts for spatial correlations between BOLD signal changes in neighboring voxels implemented in the REST toolbox (http://restfmri.net/forum/index.php), yielding an empirically determined cluster threshold of k = 37 voxels for the bilateral amygdala and a cluster threshold of k = 125 voxels for the bilateral insula.

For the between-group analysis, we entered the disgust versus neutral contrast images into a full-factorial model to assess the main effect of group and the main effect of experimental condition (masked versus unmasked disgust) as well as a group*condition interaction effect on amygdala and insula activation. Subsequent post-hoc t-tests were conducted to clarify the direction of significant main and interaction effects.

In the next step, the relationships between insula responsiveness to masked and unmasked expressions of disgust and the subjects' scores on the social loneliness and agreeableness scales were investigated. We computed simple regression models as implemented in SPM5, using the scores as regressors to the disgust versus neutral as well as the happy versus neutral contrast images. To test whether correlations would differ for patients in comparison with controls and for masked versus unmasked faces, we computed interactions between the regressors and group status/experimental condition.

Subsequently, the mean contrast values of significant clusters within the ROIs were extracted for each participant and further analyzed with SPSS 20 (IBM, Armonk, New York). We constructed multiple regression models predicting insula responsiveness by the social loneliness and the agreeableness scores, controlling for age, gender, trait anxiety and depression level.

As patients and controls differed significantly with respect to education and intelligence, we repeated all between-group analysis using education and intelligence as covariates. As between-group effects were not smaller using the covariates, we report only the results of the original analysis.

In addition to the ROI analyses, we performed exploratory whole-brain analyses which we present as supplementary material (Table S1 to Table S6 in File S1). The whole-brain analyses were conducted at a lenient level of significance (p = 0.005 uncorrected, k = 30 voxels) in order to provide a comprehensive overview of neural correlates of social loneliness and agreeableness in patients and controls.

## Results

### Questionnaire data

The reliability (internal consistency) of the social loneliness scale was high in both the patient group (α = 0.91) and the control group (α = 0.86). The sum score of social loneliness was significantly higher for the patient group, and patients showed a significantly higher within-group variance (patients: M = 36.83, SD = 12.93; controls: M = 23.88, SD = 6.56; Levene's test for homogeneity of variances: p<0.001; t = 5.42, adjusted df = 50.50, p<0.001). For the agreeableness scale, reliabilities were satisfactory (patients: α = 0.72; controls: α = 0.78). Sum scores of agreeableness were significantly lower in the patient group and variances between the patient and the control group did not differ significantly (patients: M = 31.44, SD = 5.73; controls: M = 35.23, SD = 5.57; Levene's test for homogeneity of variances: p = 0.85; t = −2.91, df = 74, p = 0.005). Social loneliness and agreeableness were negatively correlated in both the patient group (r = −0.48, p = 0.003) and the control group (r = −0.39, p = 0.014).

### Subjective awareness of masked stimuli

21 subjects (9 patients and 12 controls) reported awareness of the facial stimuli presented before the neutral faces in the masked expressions sequence. In the subsequent forced choice questionnaire, 8 subjects (3 patients and 5 controls) correctly noticed that they had perceived facial expressions of disgust. Patients and controls did not differ significantly with respect to the subjective awareness of masked facial stimuli (Chi square  = 0.237, p = 0.80) or with respect to the awareness of expressions of disgust (Chi square = 0.349, p = 0.72). Subjective awareness of the facial stimuli had no significant influence on neither insula nor amygdala activation in both groups.

### fMRI data

#### Task effects

For both the masked and the unmasked expressions sequence, the task effects on bilateral insula and amygdala activation in patients and controls were assessed using the effects-of-interest F-contrast (Family wise error correction: p = 0.001; k = 50 voxels). For both sequences, we found highly significant effects of the experimental task on bilateral insula and amygdala activation in patients and controls. Table 2 and 3 present the statistical details of task effects on insula and amygdala activation for both groups and both sequences.

**Table 2:** Masked expressions sequence: task effects on bilateral insula and amygdala activation.

Brain region	Hemisphere	Cluster size	x	y	z	Z-score	p-value (FWE-corrected)
**Patients**							
Insula	left	1409	−38	12	−4	6.82	<0.00001
	right	1485	42	−6	6	6.61	<0.00001
Amygdala	left	99	−28	4	−18	4.61	<0.00001
	right	195	26	2	−20	5.34	<0.00001
**Controls**							
Insula	left	1650	−38	16	−14	7.09	<0.00001
	right	1563	42	−8	−4	6.99	<0.00001
Amygdala	left	225	−22	0	−26	6.24	<0.00001
	right	179	26	−2	−28	7.49	<0.00001

Task effects of the masked expressions paradigm on insula and amygdala activation in patients and controls as assessed by the effects-of-interest F-contrast (Family wise error correction: p = 0.05; k = 50 voxels).

**Table 3:** Unmasked expressions sequence: task effects on bilateral insula and amygdala activation.

Brain region	Hemisphere	Cluster size	x	y	z	Z-score	p-value (FWE-corrected)
**Patients**							
Insula	left	1532	−40	−16	4	6.88	<0.00001
	right	1447	40	−10	6	6.90	<0.00001
Amygdala	left	70	−24	0	−26	6.36	<0.00001
	right	191	26	−2	−28	5.98	<0.00001
**Controls**							
Insula	left	1607	−26	18	−14	7.10	<0.00001
	right	1508	30	22	−20	7.35	<0.00001
Amygdala	left	139	−20	−2	−26	6.69	<0.00001
	right	200	36	0	−24	6.66	<0.00001

Task effects of the masked expressions paradigm on insula and amygdala activation in patients and controls as assessed by the effects-of-interest F-contrast (Family wise error correction: p = 0.05; k = 50 voxels).

#### Between-group comparisons

**Figure 2:**
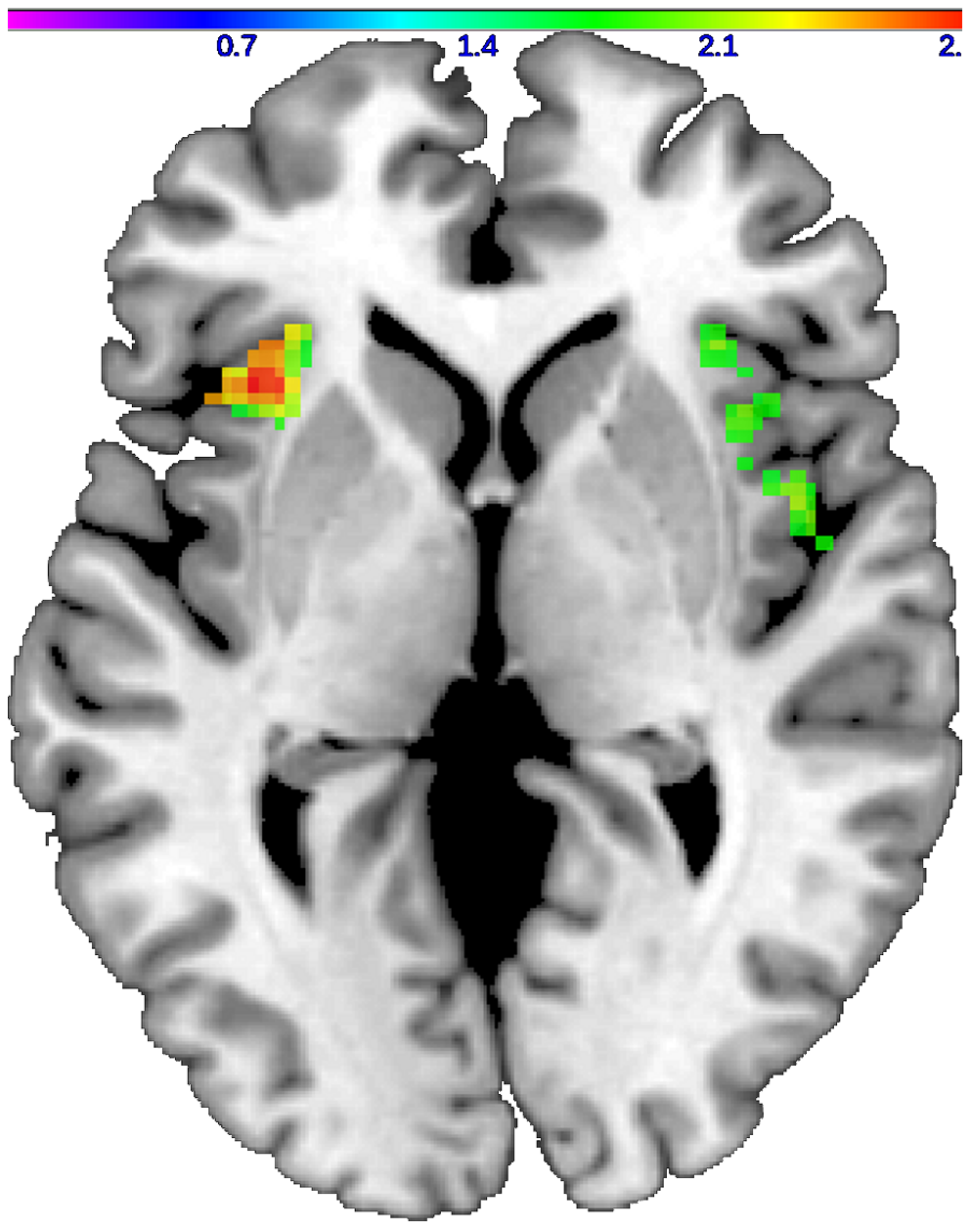
Two-sample t-test comparing insula activation in patients and controls. In comparison with controls, schizophrenia patients show reduced bilateral insula activation in response to masked facial expressions of disgust (Axial view, z = 1, color bar Z-score). Visualization was performed using a standard anatomical template from the MRIcron toolbox (www.mricro.com/mricron).

**Figure 3:**
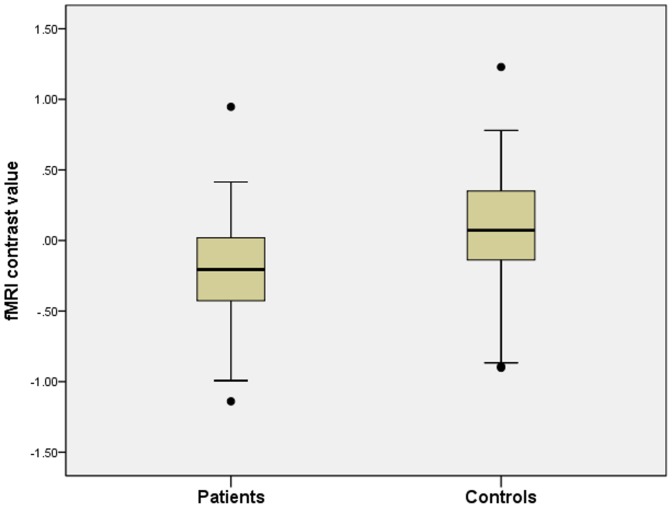
Boxplot illustrating the distribution of left insula activation values in patients and controls. Schizophrenia patients show overall reduced insula activation levels in comparison with the control group. The distribution is similar for the right insula.

The group*condition ANOVA revealed a significant main effect of condition on bilateral insula activation as subjects showed higher insula recruitment during the processing of unmasked expressions of disgust (left peak voxel xyz: −46 2 4, cluster size: 223, Z-score  = 3.43, p = 0.0003; right peak voxel xyz: 50 12 −4, cluster size: 297, Z-score  = 3.02, p = 0.001). Group status had no significant main effect on insula activation but the interaction between group and experimental condition on bilateral insula activation was significant (left peak voxel xyz: −42 18 2, cluster size: 179, Z-score  = 2.89, p = 0.002; right peak voxel xyz: 38 16 4, cluster size: 174, Z-score  = 2.59, p = 0.005). Subsequent post hoc t-tests showed reduced bilateral insula activation in patients during the processing of masked expressions of disgust (left peak voxel xyz: −36 20 2, cluster size: 174, Z-score  = 2.84, p = 0.002; right peak voxel xyz: 48 2 4, cluster size: 201, Z-score  = 2.31, p = 0.011) (Fig. 2). Figure 3 presents a boxplot illustrating the distribution of insula activation values for the left insula in patients and controls. The boxplot shows that patients exhibit overall reduced insula activation levels in comparison with controls. During the processing of unmasked expressions of disgust, we found no significant between-group difference in insula activation.

Choosing the amygdala as region of interest, the group*condition ANOVA revealed no significant main effects of group and experimental condition. Furthermore, the group*condition interaction effect on amygdala activation was not significant.

#### Correlational Analyses: social loneliness

**Figure 4:**
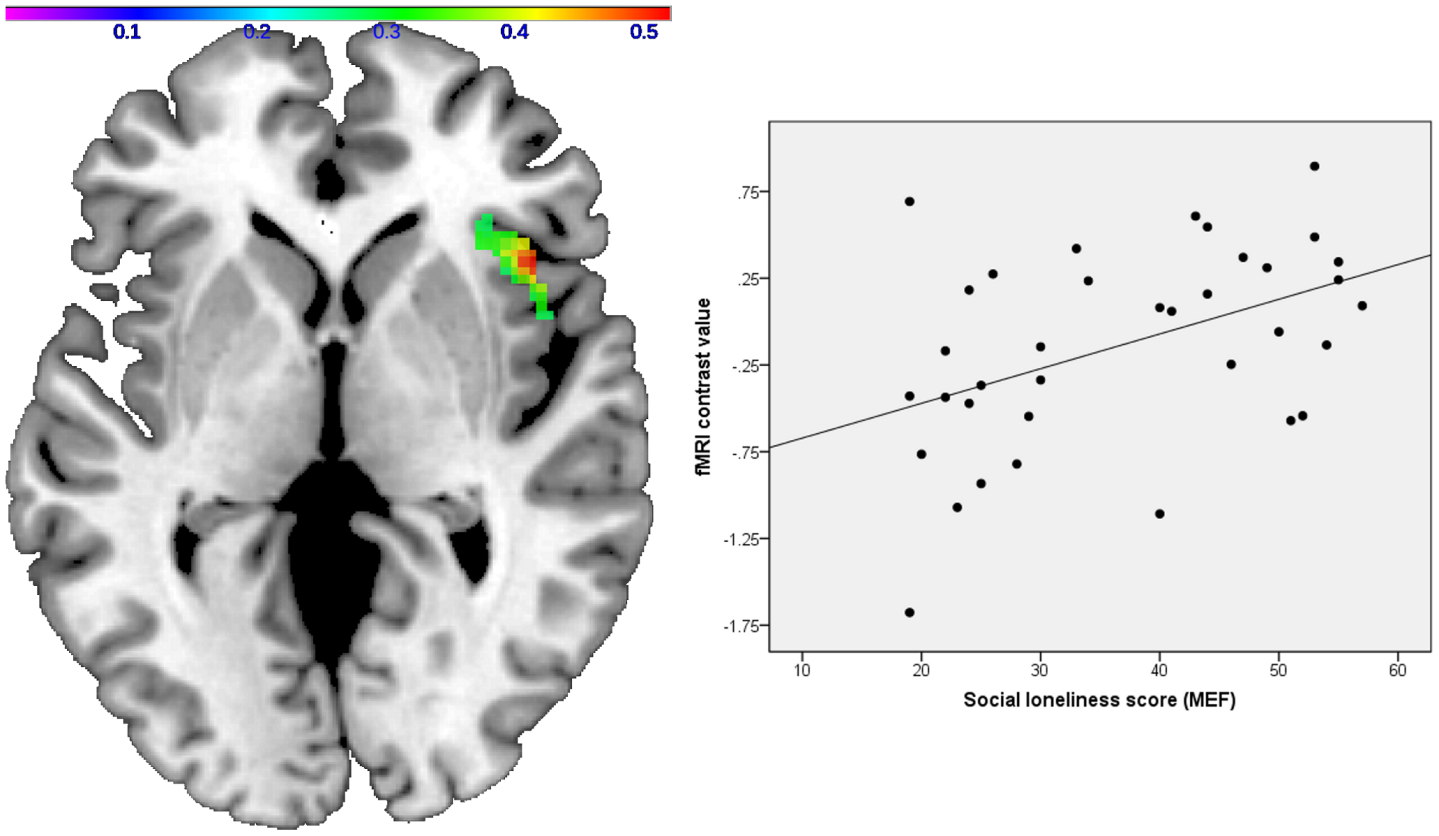
Correlation between insula activation and social loneliness in patients. In schizophrenia patients, right insula responsiveness to masked expressions of disgust is positively correlated with social loneliness (Axial view, z = 0, color bar correlation coefficient r). The scatter plot displays the correlation between the social loneliness scale scores and the mean cluster activation values (r = 0.45). Visualization was performed using a standard anatomical template from the MRIcron toolbox (www.mricro.com/mricron).

For masked disgusted versus neutral expressions, the regression analysis conducted with SPM5 yielded a significant positive correlation between the social loneliness score and right insula activation in the patient sample (peak voxel xyz: 44 20 0, cluster size: 454, Z-score  = 3.26, p = 0.0006, r = 0.45) (Fig. 4). In the subsequent multiple regression analysis predicting the mean activation of the significant cluster by the social loneliness score, Beck Depression Inventory score, State-Trait-Anxiety-Inventory trait score, age and gender, the social loneliness score was the only significant predictor (β = 0.45, t = −2.38, df = 35, p = 0.024). For controls, the social loneliness score was not significantly correlated with insula responsiveness to masked disgust versus neutral expressions. An interaction analysis confirmed that the correlation between social loneliness and insula responsiveness was indeed stronger in the patient group (peak voxel xyz: 46 22 −2, cluster size: 357, Z-score  = 2.81, p = 0.002).

For the unmasked disgust versus neutral contrast, there was no significant correlation between insula activation and the social loneliness score in both groups. For the patient group, an interaction analysis confirmed that the correlation between social loneliness and insula activation was stronger for masked expressions of disgust (peak voxel xyz: 28 14 −20, cluster size: 254, Z-score  = 4.25, p = 0.00001).

As for our control condition, the correlational analysis yielded no significant results. We found no positive or negative correlations between insula responsiveness to masked or unmasked happy versus neutral faces and the social loneliness score in both groups.

#### Correlational Analyses: agreeableness

**Figure 5:**
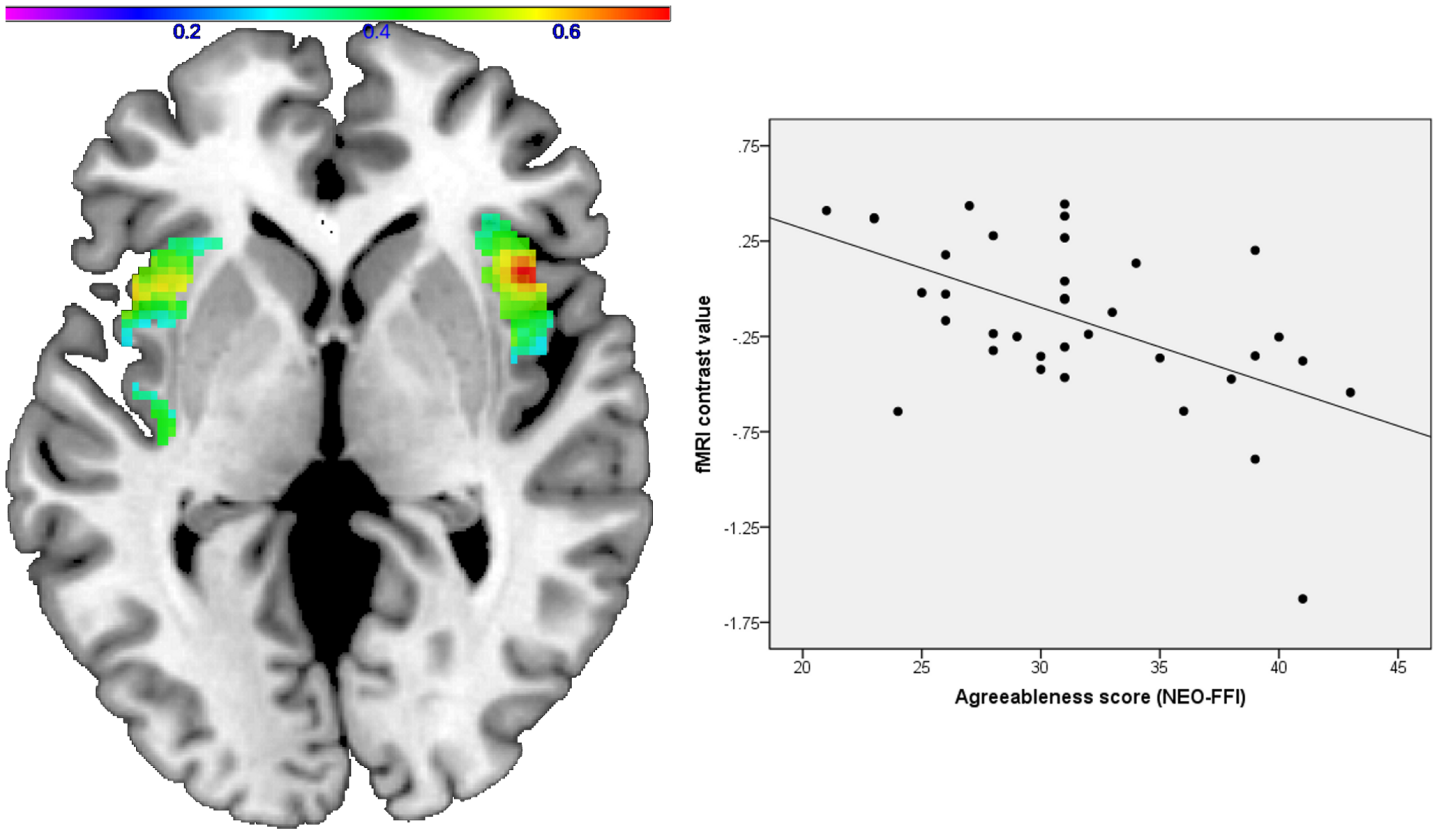
Correlation between insula activation and agreeableness in patients. In schizophrenia patients, bilateral insula responsiveness to masked expressions of disgust is negatively correlated with agreeableness (Axial view, z = 0, color bar correlation coefficient r). The scatter plot displays the correlation between the agreeableness scale scores and the mean cluster activation values for the left insula (r = −0.55). Visualization was performed using a standard anatomical template from the MRIcron toolbox (www.mricro.com/mricron).

The agreeableness score was negatively associated with bilateral insula activation to masked expressions of disgust in the patient sample (right peak voxel xyz: 42 18 0, cluster size: 842, Z-score  = 4.86, p<0.00001, r = −0.53; left peak voxel xyz: −46 12 −6, cluster size: 939, Z-score  = 3.94, p = 0.00004, r = −0.55) (Fig. 5). A multiple regression analysis showed that agreeableness was the only significant predictor of insula activation (right insula: β = −0.54, t = −3.19, df = 35, p = 0.003; left insula: β = −0.52, t = −3.15, df = 35, p = 0.004). For controls, there was also a negative correlation between the agreeableness score and bilateral insula response to masked expressions of disgust (right peak voxel xyz: 38 4 2, cluster size: 403, Z-score  = 3.02, p = 0.001, r = −0.41; left peak voxel xyz: −28 28 8, cluster size: 127, Z-score  = 2.71, p = 0.003, r = −0.36). Agreeableness was the only significant predictor of insula activation in controls (right insula: β = −0.45, t = −2.40, df = 39, p = 0.022; left insula: β = −0.36, t = −1.90, df = 39, p = 0.070). As confirmed by an interaction analysis, the negative association between agreeableness and insula responsiveness to masked disgusted faces was stronger in the patient group (right peak voxel xyz: 44 18 2, cluster size: 181, Z-score  = 2.92, p = 0.002, left peak voxel xyz: −28 16 4, cluster size: 131, Z-score  = 2.37, p = 0.009).

For the unmasked disgust versus neutral contrast, agreeableness was negatively correlated with bilateral insula activation in the patient sample (right peak voxel xyz: 42 18 −8, cluster size: 136, Z-score  = 2.78, p = 0.003; left peak voxel xyz: −26 26 8, cluster size: 170, Z-score  = 2.98, p = 0.001, r = −0.40). In the subsequent multiple regression analysis, agreeableness was the only significant predictor of left insula activation (β = −0.37, t = −2.05, df = 35, p = 0.049). In controls, agreeableness was also negatively correlated with bilateral insula response to unmasked expressions of disgust (right peak voxel xyz: 42 −18 4, cluster size: 283, Z-score  = 3.06, p = 0.001, r = −0.43; left peak voxel xyz: −36 4 −6, cluster size: 574, Z-score  = 3.00, p = 0.001, r = −0.41). Controlling for BDI score, trait anxiety, age and gender, the association between insula response to unmasked disgust and agreeableness was no more significant (p>0.1). Instead, a strong association between neural response and BDI score emerged in controls (left insula: β = 0.41, t = 2.44, df = 39, p = 0.020; right insula: β = 0.35, t = 2.03, df = 39, p = 0.050). An interaction analysis showed that the correlation between insula response to unmasked disgust and agreeableness was stronger for controls than for patients (right peak voxel xyz: 44 −2 −8, cluster size: 389, Z-score  = 3.16, p = 0.0008, left peak voxel xyz: −36 14 −10, cluster size: 373, Z-score  = 2.77, p = 0.003).

In patients, the negative correlation between insula response and agreeableness was stronger for masked than for unmasked expressions of disgust (right peak voxel xyz: 40 −2 −6, cluster size: 564, Z-score  = 3.49, p = 0.0003, left peak voxel xyz: −44 14 0, cluster size: 539, Z-score  = 2.99, p = 0.001). In controls, there was no significant interaction between the agreeableness score and experimental condition.

Using the masked and unmasked happy versus neutral contrast images in the correlational analyses yielded no significant positive or negative association between insula responsiveness and agreeableness in the patient group. In controls, we found a negative correlation between right insula response to masked happy faces and agreeableness (peak voxel xyz: 34 14 −10, cluster size: 183, Z-score  = 2.74, p = 0.003, r = −0.39). However, this correlation was no more significant after controlling for BDI score, trait anxiety, age and gender (p = 0.87).

## Discussion

Disgust has been dubbed “the forgotten emotion of psychiatry” [42]. The present study has revealed new insights into the relevance of this emotion in schizophrenia. Our hypothesis of lower insula activation during the processing of facial expressions of disgust in schizophrenia patients compared with healthy controls was partially confirmed. We found reduced insula activation in patients during the processing of micro-expressions of disgust. This indicates that patients might have a reduced perceptual sensitivity to covert facial expressions of disgust. On the contrary, no between-group difference was found for the processing of overt expressions of disgust. This result is in contrast to the findings of Phillips et al. [14] who found reduced insula activation in response to overt expressions of disgust in schizophrenia patients. Phillips et al. used an emotion recognition paradigm which is cognitively more demanding than a passive viewing task. Under conditions of cognitive load, the processing of overt facial expressions of disgust might also be impaired in patients.

For the amygdala, no between-group difference emerged for the processing of either micro-expressions or macro-expressions of disgust. This is not surprising, as the amygdala seems not primarily involved in the processing of disgust [13].

With respect to the questionnaire data, patients had significantly higher scores on the social loneliness scale and lower scores on the agreeableness scale than controls. This parallels earlier findings of increased loneliness [43] and reduced agreeableness [44] in schizophrenia. The correlational analyses confirmed our hypothesis that insula responsiveness to expressions of disgust would be associated with social alienation in patients. In our patient sample, insula activation to masked expressions of disgust was positively correlated with social loneliness and insula activation to masked and unmasked expressions of disgust was negatively correlated with agreeableness. These associations were not confounded by depressivity, trait anxiety, age and gender. In our patient sample, the correlations between our indicators of social alienation and insula responsiveness were stronger using masked expressions of disgust. Furthermore, these associations were stronger in patients than in controls. As for our control condition of masked and unmasked expressions of happiness, we found no significant correlations between insula responsiveness and our variables of social alienation. Taken together, these results indicate that insula responsiveness to micro-expressions of disgust might be a specific predictor of social alienation in schizophrenia. Given that the insula responds to experiences of social rejection and social pain [45], this finding is plausible: Patients experiencing higher degrees of social alienation seem to exhibit an increased insular sensitivity to subtle signals of social rejection.

It should be taken into account that schizophrenia patients often face stigmatization and social rejection due to the disease [22]. Taking this into consideration, patients' overall reduced insula activation in response to facial micro-expressions of disgust warrants an interpretation beyond the deficit perspective. Reduced perceptual sensitivity to subtle signals of social rejection might protect patients from social alienation. If this is the case, clinical interventions like the training of micro-expression recognition in schizophrenia [46] might have detrimental effects on patients' social sensitivities and social lives. Further studies on the processing of micro-expressions of disgust in schizophrenia are needed to clarify this issue. Our finding seems also interesting in the context of expressed emotion research in schizophrenia. There is clear evidence for a robust relationship between patients' exposure to relatives' overt negative emotions and psychiatric relapse [47]. Patients with a high sensitivity for even minimal interpersonal signals of rejection and disapproval could be especially at risk for decompensation and relapse. These patients might seek refuge in social withdrawal and isolation.

Importantly, we also found a negative association between the personality trait of agreeableness and insula responsiveness to masked and unmasked disgust in healthy controls. Whereas patients showed a stronger association between agreeableness and insula activation during the processing of masked disgust faces, the opposite result emerged during the processing of unmasked disgust faces. Here, the negative correlation between insula responsiveness and agreeableness was even stronger in controls than in patients. This finding requires careful interpretation, as the correlation was confounded by depressivity in controls. However, in view of our correlational findings for schizophrenia patients and healthy controls it appears that insula responsiveness to micro-expressions of disgust might be in general a neural marker of agreeableness. Future research should follow up on this hypothesis.

In our control group, we found no significant correlation between insula responsiveness to expressions of disgust and social loneliness. However, this lack of a finding might be due to reduced within-group variance in controls, as loneliness is more pronounced in clinical populations. It might be promising to investigate whether the positive correlation between insula responsiveness to disgust and social loneliness is characteristic for schizophrenia patients or also emerges in other patient groups. Future research could also test the relationship between insula responsiveness to disgust faces and other non-clinical measures of social integration in healthy controls. In particular, subjective and objective measures of experienced discrimination and social rejection might offer valuable insights. Recent studies have shown that the insula responsiveness to social rejection in a social exclusion paradigm is related to interindividual differences in discrimination-related distress [48] and attachment style [49].

The present study showed that decreased insula responsiveness to disgust in patients is related to lower degrees of social alienation. It would be important to interpret these findings in light of a general theory of insula dysfunction in schizophrenia. A growing body of research suggests a crucial role of the insula in the neuropathology of the disease. There is consistent evidence that insula grey matter volume is substantially decreased in schizophrenia patients [50]. The insula is a widely interconnected brain structure with extensive connections to sensory, somatosensory, limbic and prefrontal regions. It has been shown to fulfill a central function in the integration of sensory, interoceptive and emotional information [16], [50], [51]. According to a recent model of insula function, the insula seems to be involved in emotional awareness [51]. Impaired insula function in schizophrenia has been related to general deficits in self-awareness and in the neural representation of the self which encompass both impairments in the processing of affective experience and in the processing of pain [16]. Interestingly, asymbolia for physical pain has been described as a possible trait marker of schizophrenia [52]. In this light, our finding of reduced insula responsiveness to disgusted faces might hint to a certain asymbolia for social pain.

Future research should investigate the role of insula-limbic connectivity in schizophrenia. In healthy subjects, the insula shows extensive connections with the amygdala and the anterior cingulate cortex [16]. This insula-limbic connectivity might be reduced in patients and thus further account for deficits in social cognition.

To our knowledge, this is the first neuroimaging study investigating the processing of micro-expressions of disgust in schizophrenia and one of few studies dedicated to the social dimension of this emotion. Despite these strengths, some limitations of the study should be noted. First of all, using a passive viewing task we had no control as to whether all subjects equally attended to the presented stimuli. This is important as schizophrenia patients often suffer from amotivation. Future research might test experimental paradigms which are more engaging but equally simple with respect to cognitive demand. Another important limitation of the study consists in the restricted ecological validity of our paradigm. We used a masking procedure to mimic rapid changes in facial expressions. Anyhow, this approach represents only a crude simulation of facial micro-expressions as mimicry in everyday communication occurs in a more fluid and less distinct way. Moreover, the backward masking procedure raises questions about the role of early visual processing deficits in schizophrenia. Schizophrenia patients show impairments in visual backward masking [53], [54] and these deficits might account for our finding of insular hypoactication during the processing of masked disgust faces. However, the patient group did not differ significantly from the control group with respect to the subjective awareness for the masked stimuli and awareness had no significant influence on insula activation. A further potential problem related to our experimental paradigm may consist in the relatively short presentation time of the facial emotion conditions. As each condition was presented for a total of only 66 seconds, our data basis is limited. However, this concern is attenuated by the fact that we found similar correlations of agreeableness with insula responsiveness to facial disgust for both the masked and the unmasked presentation condition in the patient sample. Moreover, negative correlations of agreeableness with insula responsiveness to covert facial expressions of disgust were found for patients *and* controls. Nevertheless, future studies are clearly needed to replicate our between-group and correlation findings before strong conclusions can be drawn. In particular, it would be important to investigate whether the same results are obtained using an event-related design. Another limitation could be seen in the high neuroleptic medication of the patient group. Replicating the study with unmedicated patients would be necessary to assure that our finding of insular hypoactivation to masked disgust in the patient group is not an effect of neuroleptic medication. Furthermore, it should be acknowledged that our correlational analyses do not justify conclusions about the direction of the relationship between social alienation and the neural responsiveness to expressions of disgust. Long-term studies could provide new evidence on how the neural responsiveness to disgust develops in the course of the disease.

## Supporting Information

File S1 **Supporting information tables S1 – S6. Results of supplementary whole-brain analyses.**
(DOC)
