# Supplementary material for: Social Alienation in Schizophrenia Patients: Association with Insula Responsiveness to Facial Expressions of Disgust
Source: PLoS One. 2014 Jan 22;9(1):e85014. doi: 10.1371/journal.pone.0085014 (PMC3898910; doi:10.1371/journal.pone.0085014)
Supplement: File S1 — Supporting information tables S1 – S6. Results of supplementary whole-brain analyses. (DOC) [file pone.0085014.s001.doc]

**Table S1:**

Differences between schizophrenia patients and control subjects in cerebral response to *masked* disgust versus neutral facial expressions: Results of whole-brain two-sample t-tests conducted at p<.005, uncorrected, k=30 voxels. The table shows areas of hyperactivation for patients versus control subjects and vice versa.

| Anatomical region | BA | Side | Cluster size | x | y | z | Z-score | p-value (uncorr.) |
| --- | --- | --- | --- | --- | --- | --- | --- | --- |
| **Controls > Patients** | | | | | | | | |
| Middle frontal gyrus | 6 | L | 58 | -40 | 8 | 56 | 3.56 | .0002 |
| Insula, inferior frontal gyrus | 13, 47 | L | 38 | -32 | 30 | 0 | 2.97 | .001 |
| **Patients > Controls** | | | | | | | | |
| Middle occipital gyrus | 18 | L | 62 | -12 | -102 | 8 | 3.46 | .0003 |
| Cingulate gyrus, medial frontal gyrus | 6, 24 | R | 42 | 16 | -4 | 54 | 3.43 | .0003 |

Coordinates are given in MNI space and mark the maximal point of activation.

**Table S2:**

Differences between schizophrenia patients and control subjects in cerebral response to *unmasked* disgust versus neutral facial expressions: Results of whole-brain two-sample t-tests conducted at p<.005, uncorrected, k=30 voxels. The table shows areas of hyperactivation for patients versus control subjects and vice versa.

| Anatomical region | BA | Side | Cluster size | x | y | z | Z-score | p-value (uncorr.) |
| --- | --- | --- | --- | --- | --- | --- | --- | --- |
| **Controls > Patients** | | | | | | | | |
| Cingulate gyrus | 24 | L | 41 | -6 | -6 | 30 | 3.26 | .0006 |
| **Patients > Controls** | | | | | | | | |
| Parahippocampal gyrus | - | L | 63 | -30 | -6 | -24 | 3.78 | .0001 |
| Anterior Cingulate, superior frontal gyrus, medial frontal gyrus | 9, 32 | R | 32 | 18 | 40 | 20 | 3.71 | .0001 |
| Caudate | - | L | 35 | -10 | 10 | 16 | 3.61 | .0002 |
| Parahippocampal gyrus | - | R | 48 | 30 | -2 | -28 | 3.42 | .0003 |
| Parahippocampal gyrus | - | L | 35 | -30 | -28 | -16 | 3.35 | .0004 |
| Posterior cingulate | 30 | R | 51 | 18 | -56 | 10 | 3.28 | .0005 |
| Posterior Cingulate, parahippocampal gyrus | 29, 30 | R | 43 | 12 | -42 | 4 | 3.16 | .0008 |
| Middle temporal gyrus | 19, 39 | R | 73 | 48 | -66 | 14 | 3.08 | .001 |

Coordinates are given in MNI space and mark the maximal point of activation.

**Table S3:**

Positive correlation between patients’ social loneliness score and cerebral response to *masked* disgust versus neutral facial expressions. Results of a whole brain regression analysis conducted at p<.005, uncorrected, k=30 voxels. For controls, there were no regions showing activity associated with social loneliness at this level of significance.

| Anatomical region | BA | Side | Cluster size | x | y | z | Z-score | p-value (uncorr.) |
| --- | --- | --- | --- | --- | --- | --- | --- | --- |
| Superior frontal gyrus, medial frontal gyrus | 8 | R | 103 | 8 | 34 | 46 | 3.59 | .0002 |
| Anterior cingulate, superior frontal gyrus, medial frontal gyrus | 9, 10, 32, 42 | R | 130 | 10 | 48 | 8 | 3.49 | .0002 |
| Insula | 13 | R | 44 | 32 | 0 | 14 | 3.43 | .0003 |
| Middle frontal gyrus, superior frontal gyrus | 10, 11 | R | 71 | 42 | 56 | -4 | 3.36 | .0004 |
| Thalamus | - | R | 56 | 12 | -14 | 6 | 3.31 | .0005 |
| Insula, precentral gyrus, inferior frontal gyrus | 13, 44, 45, 47 | R | 309 | 46 | 18 | 4 | 3.28 | .0005 |
| Parahippocampal gyrus, inferior frontal gyrus | 34, 47 | R | 95 | 34 | -6 | -8 | 3.22 | .0006 |
| Superior frontal gyrus, medial frontal gyrus | 9 | R, L | 42 | 2 | 50 | 28 | 3.16 | .0008 |
| Caudate | - | L | 31 | -8 | 8 | 8 | 3.15 | .0008 |
| Middle temporal gyrus, superior temporal gyrus | 13, 39 | R | 72 | 44 | -60 | 8 | 3.10 | .001 |
| Lentiform nucleus | - | R | 30 | 26 | -10 | 6 | 2.93 | .002 |
| Fusiform gyrus, inferior occipital gyrus, lingual gyrus, middle occipital gyrus | 19 | R | 30 | 36 | -78 | -14 | 2.91 | .002 |

Coordinates are given in MNI space and mark the maximal point of activation.

**Table S4:**

Positive correlation between subjects’ social loneliness score and cerebral response to *unmasked* disgust versus neutral facial expressions. Results of a whole brain regression analysis conducted at p<.005, uncorrected, k=30 voxels.

| Anatomical region | BA | Side | Cluster size | x | y | z | Z-score | p-value (uncorr.) |
| --- | --- | --- | --- | --- | --- | --- | --- | --- |
| **Controls** | | | | | | | | |
| Middle occipital gyrus | 18, 19 | L | 30 | -26 | -88 | 4 | 3.58 | .0002 |
| Anterior cingulate, medial frontal gyrus | 9, 32 | L | 50 | -22 | 34 | 22 | 3.15 | .0008 |
| Superior frontal gyrus, medial frontal gyrus | - | R | 33 | 14 | 56 | 22 | 3.11 | .0009 |
| **Patients** | | | | | | | | |
| Middle temporal gyrus, precuneus | 39 | R | 196 | 32 | -60 | 20 | 4.45 | <.0001 |
| Cingulate gyrus | 32 | R | 60 | 22 | 20 | 36 | 3.70 | .0001 |
| Insula | 13 | L | 78 | -32 | 2 | 20 | 3.46 | .0003 |
| Cingulate gyrus | - | R | 42 | 14 | -24 | 30 | 3.26 | .0006 |
| Cingulate gyrus, superior frontal gyrus, medial frontal gyrus | 24, 32 | L | 46 | -16 | 16 | 38 | 3.22 | .0006 |

Coordinates are given in MNI space and mark the maximal point of activation.

**Table S5:**

Negative correlation between subjects’ agreeableness score and cerebral response to *masked* disgust versus neutral facial expressions. Results of a whole brain regression analysis conducted at p<.005, uncorrected, k=30 voxels.

| Anatomical region | BA | Side | Cluster size | x | y | z | Z-score | p-value (uncorr.) |
| --- | --- | --- | --- | --- | --- | --- | --- | --- |
| **Controls** | | | | | | | | |
| Precentral gyrus, supramarginal gyrus, middle frontal gyrus, postcentral gyrus, medial frontal gyrus | 1, 2, 3, 4, 5, 6, 40 | R | 881 | 12 | -36 | 56 | 3.82 | <.0001 |
| Insula, precentral gyrus, inferior frontal gyrus, middle frontal gyrus | 9, 10, 11, 13, 44, 45, 46 | R | 921 | 50 | 12 | 12 | 3.80 | <.0001 |
| Precentral gyrus, middle frontal gyrus | 6 | L | 33 | -34 | -12 | 44 | 3.60 | .0002 |
| Postcentral gyrus | 1, 2, 3 | L | 134 | -28 | -36 | 68 | 3.47 | .0003 |
| Middle frontal gyrus, superior frontal gyrus | 6 | R | 113 | 24 | -4 | 68 | 3.46 | .0003 |
| Precentral gyrus, postcentral gyrus | 1, 3, 4 | L | 61 | -40 | -26 | 58 | 3.42 | .0003 |
| Supramarginal gyrus | 40 | L | 67 | -44 | -46 | 40 | 3.33 | .0004 |
| Precuneus, paracentral lobule | 3, 4, 5 | L | 51 | -16 | -40 | 52 | 3.31 | .0005 |
| Medial frontal gyrus | 6 | L | 37 | -2 | -22 | 66 | 3.29 | .0005 |
| Thalamus | - | R | 50 | 16 | -16 | -4 | 3.21 | .0007 |
| Insula | - | R | 40 | 36 | 4 | 0 | 3.18 | .0007 |
| Middle temporal gyrus | 37 | R | 47 | 54 | -60 | 0 | 3.15 | .0008 |
| Precentral gyrus, postcentral gyrus, inferior parietal lobule | 1, 2, 3, 4 | L | 155 | -46 | -32 | 46 | 3.14 | .0008 |
| Middle frontal gyrus, medial frontal gyrus | 6 | L | 32 | -20 | -8 | 58 | 3.14 | .0008 |
| Middle frontal gyrus, superior frontal gyrus | 8 | R | 62 | 30 | 18 | 44 | 3.10 | .001 |
| Superior frontal gyrus, medial frontal gyrus | 8 | R | 48 | 10 | 30 | 44 | 3.07 | .001 |
| Lentiform nucleus | - | L | 37 | -10 | -6 | -10 | 3.06 | .001 |
| **Patients** | | | | | | | | |
| Insula, parahippocampal gyrus, superor temporal gyrus, precentral gyrus, inferior frontal gyrus, middle frontal gyrus, superior frontal gyrus, postcentral gyrus | 1, 2, 3, 4, 6, 8, 9, 13, 28, 34, 38, 44, 45, 46, 47 | R | 2946 | 42 | 18 | 0 | 4.86 | <.0001 |
| Insula, superior temporal gyrus, supramarginal gyrus, inferior parietal lobule | 13, 40, 42 | L | 187 | -44 | -38 | 26 | 4.57 | <.0001 |
| Insula, superior temporal gyrus, precentral gyrus, inferior frontal gyrus | 13, 22, 38, 44, 45, 47 | L | 777 | -48 | 14 | -4 | 4.44 | <.0001 |
| Anterior cingulate, cingulate gyrus, middle frontal gyrus, medial frontal gyrus, superior frontal gyrus, | 6, 8, 24, 32 | R, L | 800 | -10 | 6 | 52 | 4.25 | <.0001 |
| Middle frontal gyrus, superior frontal gyrus, medial frontal gyrus | 9, 10, 46 | L | 532 | -42 | 50 | 16 | 4.12 | <.0001 |
| Superior frontal gyrus, medial frontal gyrus | 6 | L | 228 | -8 | -2 | 70 | 3.90 | <.0001 |
| Inferior frontal gyrus, middle frontal gyrus, superior frontal gyrus | 10, 44, 46, 47 | R | 600 | 44 | 50 | 18 | 3.79 | <.0001 |
| Insula, inferior parietal lobule, postcentral gyrus | 13 | R | 83 | 44 | -26 | 24 | 3.71 | .0001 |
| Insula, middle temporal gyrus, superior temporal gyrus | 13, 22 | L | 226 | -28 | -30 | 8 | 3.64 | .0001 |
| Middle temporal gyrus, superior temporal gyrus | 21, 38 | R | 45 | 48 | 4 | -30 | 3.59 | .0002 |
| Fusiform gyrus, parahippocampal gyrus, lingual gyrus | 19, 37 | L | 164 | -24 | -54 | -14 | 3.56 | .0002 |
| Middle occipital gyrus, middle temporal gyrus | 37, 39 | R | 112 | 40 | -62 | 6 | 3.56 | .0002 |
| Supramarginal gyrus, inferior parietal lobule, postcentral gyrus | 2, 40 | R | 153 | 58 | -38 | 32 | 3.56 | .0002 |
| Precentral gyrus, middle frontal gyrus | 6 | L | 183 | -36 | 2 | 52 | 3.54 | .0002 |
| Postcentral gyrus | 3, 40 | L | 42 | -26 | -38 | 56 | 3.53 | .0002 |
| Fusiform gyrus, inferior occipital gyrus, middle occipital gyrus, inferior temporal gyrus | 19, 37 | R | 111 | 42 | -76 | -6 | 3.45 | .0003 |
| Insula, middle temporal gyrus, superior temporal gyrus | 13, 22, 42 | R | 107 | 52 | -36 | 6 | 3.39 | .0003 |
| Precentral gyrus | 4, 6 | R | 92 | 20 | -22 | 72 | 3.37 | .0004 |
| Supramarginal gyrus | - | L | 58 | -30 | -54 | 30 | 3.36 | .0004 |
| Fusiform gyrus, inferior temporal gyrus, middle occipital gyrus | 37 | L | 62 | -50 | -60 | -22 | 3.35 | .0004 |
| Anterior cingulate, cingulate gyrus, medial frontal gyrus | 9, 24, 32, 33 | R, L | 155 | 4 | 18 | 22 | 3.34 | .0004 |
| Precentral gyrus, inferior frontal gyrus, middle frontal gyrus | 6, 9 | L | 89 | -38 | -4 | 32 | 3.33 | .0004 |
| Middle temporal gyrus | - | L | 51 | -48 | -32 | -16 | 3.30 | .0005 |
| Middle temporal gyrus | - | R | 120 | 50 | -44 | -10 | 3.27 | .0005 |
| Precuneus, inferior parietal lobule, superior parietal lobule | 7, 40 | L | 87 | -24 | -56 | 52 | 3.27 | .0005 |
| Middle occipital gyrus, inferior temporal gyrus, middle temporal gyrus | 19, 21, 37 | L | 118 | -52 | -64 | -8 | 3.25 | .0006 |
| Inferior occipital gyrus, middle occipital gyrus | 18, 19 | L | 99 | -40 | -90 | -12 | 3.25 | .0006 |
| Fusiform gyrus, inferior occipital gyrus, middle occipital gyrus, lingual gyrus | 17, 18, 19 | L | 45 | -20 | -88 | -16 | 3.18 | .0007 |
| Inferior parietal lobule, postcentral gyrus | 1, 2, 3, 40 | R | 112 | 48 | -40 | 48 | 3.14 | .0008 |
| Insula, parahippocampal gyrus, middle temporal gyrus, superior temporal gyrus | 13, 21, 38 | L | 88 | -42 | 0 | -12 | 3.13 | .0009 |
| Postcentral gyrus | 1, 2, 3 | R | 81 | 54 | -20 | 52 | 3.06 | .001 |
| Insula | - | R | 50 | 40 | -18 | -12 | 3.02 | .001 |
| Caudate | - | R, L | 56 | -8 | 6 | 14 | 3.01 | .001 |
| Angular gyrus, supramarginal gyrus, inferior parietal lobule | 40 | R | 31 | 34 | -50 | 32 | 2.98 | .001 |

Coordinates are given in MNI space and mark the maximal point of activation.

**Table S6:**

Negative correlation between subjects’ agreeableness score and cerebral response to *unmasked* disgust versus neutral facial expressions. Results of a whole brain regression analysis conducted at p<.005, uncorrected, k=30 voxels.

| Anatomical region | BA | Side | Cluster size | x | y | z | Z-score | p-value (uncorr.) |
| --- | --- | --- | --- | --- | --- | --- | --- | --- |
| **Controls** | | | | | | | | |
| Precentral gyrus, postcentral gyrus | 2 | L | 51 | -40 | -22 | 34 | 4.07 | <.0001 |
| Insula, transverse temporal gyrus, middle temporal gyrus, superior temporal gyrus, precentral gyrus | 6, 13, 21, 22, 29, 41 | R | 281 | 38 | -26 | 10 | 4.03 | <.0001 |
| Precuneus, superior parietal lobule, paracentral lobule, postcentral gyrus | 4, 7 | R | 221 | 16 | -60 | 62 | 3.78 | <.0001 |
| Insula | 13 | R | 38 | 30 | -12 | 16 | 3.75 | <.0001 |
| Insula, superior temporal gyrus | 13, 29 | L | 56 | -44 | -26 | 16 | 3.74 | <.0001 |
| Insula | - | L | 36 | -28 | 2 | 24 | 3.62 | .0001 |
| Insula | - | L | 48 | -26 | -18 | 22 | 3.60 | .0002 |
| Precuneus, angular gyrus, supramarginal gyrus | - | L | 61 | -22 | -60 | 30 | 3.54 | .0002 |
| Precentral gyrus, postcentral gyrus, inferior parietal lobule | 3, 4, 40 | R | 62 | 36 | -36 | 50 | 3.53 | .0002 |
| Middle frontal gyrus, superior frontal gyrus, medial frontal gyrus | 10, 11 | R | 78 | 20 | 54 | -4 | 3.46 | .0003 |
| Precentral gyrus, middle frontal gyrus | 6 | L | 171 | -30 | -12 | 54 | 3.41 | .0003 |
| Middle temporal gyrus, superior temporal gyrus | 21, 22 | R | 72 | 58 | 0 | -8 | 3.37 | .0004 |
| Superior frontal gyrus, medial frontal gyrus | - | R | 44 | 14 | 16 | 52 | 3.36 | .0004 |
| Supramarginal gyrus, inferior parietal lobule | 40 | R | 36 | 52 | -50 | 24 | 3.35 | .0004 |
| Inferior frontal gyrus | 45, 47 | R | 45 | 56 | 24 | 4 | 3.33 | .0004 |
| Precuneus, superior parietal lobule | 7 | R, L | 326 | -10 | -68 | 62 | 3.30 | .0005 |
| Middle frontal gyrus, superior frontal gyrus | 8 | R | 32 | 24 | 22 | 52 | 3.28 | .0005 |
| Insula | 13 | L | 87 | -32 | 4 | -2 | 3.27 | .0005 |
| Middle frontal gyrus, superior frontal gyrus | 6 | R | 234 | 26 | -4 | 68 | 3.26 | .0006 |
| Insula, middle temporal gyrus, superior temporal gyrus, precentral gyrus | 13, 21, 22, 38 | L | 164 | -52 | -12 | 4 | 3.23 | .0006 |
| Postcentral gyrus | 3, 40 | R | 33 | 20 | -40 | 58 | 3.23 | .0006 |
| Lentiform nucleus | - | L | 33 | -22 | -6 | 4 | 3.20 | .0007 |
| Superior temporal gyrus | 21, 22, 29, 41, 42 | R | 110 | 66 | -18 | 6 | 3.13 | .0009 |
| Cingulate gyrus | - | R | 38 | 10 | -16 | 34 | 3.10 | .001 |
| Middle temporal gyrus, superior temporal gyrus | 21, 22 | L | 55 | -56 | -30 | -2 | 3.07 | .001 |
| Precuneus, inferior parietal lobule | - | L | 46 | -30 | -44 | 46 | 3.07 | .001 |
| Precuneus, paracentral lobule | 5, 7 | R, L | 50 | -4 | -50 | 54 | 3.05 | .001 |
| Middle frontal gyrus, superior frontal gyrus | 9, 10 | L | 30 | -28 | 40 | 30 | 3.00 | .001 |
| **Patients** | | | | | | | | |
| Inferior frontal gyrus, middle frontal gyrus, superior frontal gyrus | 10, 46 | R | 227 | 42 | 56 | -2 | 4.61 | <.0001 |
| Precentral gyrus, cingulate gyrus, middle frontal gyrus, superior frontal gyrus | 6, 8, 24, 32 | R, L | 763 | 14 | 10 | 54 | 4.56 | <.0001 |
| Precuneus, inferior parietal lobule, paracentral lobule, medial frontal gyrus | 4, 5, 6 | R | 387 | 24 | -36 | 42 | 4.46 | <.0001 |
| Precentral gyrus, cingulate gyrus, inferior frontal gyrus, middle frontal gyrus | 6, 9, 24, 32 | L | 193 | -32 | 0 | 40 | 4.06 | <.0001 |
| Precentral gyrus, cingulate gyrus, paracentral lobule, middle frontal gyrus, superior frontal gyrus, medial frontal gyrus | 5, 6, 24 | L | 301 | -16 | -32 | 50 | 4.03 | <.0001 |
| Insula | 13 | L | 51 | -32 | 0 | 22 | 3.88 | <.0001 |
| Precentral gyrus, middle frontal gyrus | 6 | R | 93 | 26 | -14 | 44 | 3.76 | <.0001 |
| Inferior frontal gyrus, middle frontal gyrus | 9 | L | 30 | -58 | 16 | 30 | 3.68 | .0001 |
| Cingulate gyrus | 24, 32 | L | 50 | -14 | 18 | 38 | 3.58 | .0002 |
| Middle frontal gyrus | 10 | L | 91 | -36 | 54 | -4 | 3.54 | .0002 |
| Inferior temporal gyrus, middle temporal gyrus | 37 | L | 64 | -50 | -48 | -10 | 3.50 | .0002 |
| Precuneus | 7 | R | 99 | 14 | -74 | 42 | 3.46 | .0003 |
| Inferior frontal gyrus | - | L | 80 | -34 | 32 | 10 | 3.40 | .0003 |
| Middle occipital gyrus | 19 | R | 33 | 34 | -78 | 4 | 3.35 | .0004 |
| Precentral gyrus, middle frontal gyrus, superior frontal gyrus, medial frontal gyrus | 6 | R | 66 | 10 | -20 | 76 | 3.33 | .0004 |
| Inferior frontal gyrus, middle frontal gyrus | 10, 46 | L | 69 | -44 | 40 | 18 | 3.14 | .0008 |
| Middle occipital gyrus, middle temporal gyrus | - | L | 34 | -28 | -76 | 18 | 3.13 | .0009 |

Coordinates are given in MNI space and mark the maximal point of activation.
